# Practise of Immunoglobulin Replacement Therapy in Primary and Secondary Immunodeficiencies: A Single Centre Experience from Malaysia

**DOI:** 10.21315/mjms2023.30.3.10

**Published:** 2023-06-27

**Authors:** Nurul Hidayah Zahari, Intan Juliana Abd Hamid, Sharifah Azdiana Tuan Din, Ilie Fadzilah Hashim, Zarina Thasneem Zainudeen, Noorsuzana Mohd Shariff, Nor Hafizah Ahmad, Chan Yen Tay

**Affiliations:** 1 Department of Clinical Medicine, Advanced Medical and Dental Institute, Universiti Sains Malaysia, Pulau Pinang, Malaysia; 2 Primary Immunodeficiency Diseases Group, Department of Clinical Medicine, Advanced Medical and Dental Institute, Universiti Sains Malaysia, Pulau Pinang, Malaysia; 3 Health Community Department, Advanced Medical and Dental Institute, Universiti Sains Malaysia, Pulau Pinang, Malaysia; 4 Clinical Transfusion Department, National Blood Centre, Kuala Lumpur, Malaysia; 5 Pharmacy Department, Hospital Kuala Lumpur, Kuala Lumpur, Malaysia

**Keywords:** intravenous immunoglobulin, off-labelled indication, hypogammaglobulinemia, chronic inflammatory demyelinating polyneuropathy, primary immunodeficiencies

## Abstract

**Background:**

Intravenous immunoglobulin (IVIG) replacement therapy is increasingly in demand. This study focused on the characteristics of IVIG usage and associated factors toward the frequency status of IVIG among patients in Hospital Kuala Lumpur.

**Methods:**

A retrospective cross-sectional study was performed on patients who received IVIG in Hospital Kuala Lumpur. Data were extracted from the request forms for IVIG recorded in the Pharmacy Department from January 2018 until December 2019. Chi-squared test and *t*-test analysis were used for statistical analysis, and a *P*-value of < 0.05 was considered significant.

**Results:**

A total of 482 patients received IVIG in Hospital Kuala Lumpur. There were 243 (50.4%) females and 228 (47.3%) males with median age of the patients was 27 years old. The highest indications for IVIG among all patients were hypogammaglobulinemia and other deficiency states in 127 patients (26.3%). The most common indication for one-off treatment in adults was hypogammaglobulinemia and other deficiency states, 35%; whereas in paediatrics, it was Kawasaki disease, 20.3%. The highest indication for regular therapy among adult patients was chronic inflammatory demyelinating polyneuropathy (23.4%), while in paediatrics it was sepsis (31.1%). The clinical category was associated with the frequency status of IVIG usage in both adult and paediatric cohorts with *P* = 0.004 and *P* = 0.017, respectively.

**Conclusion:**

There were significant differences between the indication of one-off treatment and regular therapy among adult and paediatric patients. A national guideline on the prescription of IVIG for patients is instantly needed to help clinicians in prescribing IVIG appropriately.

## Introduction

Intravenous immunoglobulin (IVIG) is a medicinal formulation of pooled poly-speciﬁc immunoglobulin G (IgG) derived from the plasma of a thousand or more healthy individuals (1–3). IVIG is one of the plasma-derived medicinal products (PDMP) under the fractionation programme (4). According to the World Health Organization (WHO), only 55 of 171 reporting countries have PDMP through the fractionation of plasma collected in the reporting country (5). Approximately 25.6 million litres of plasma from 39 reporting nations were fractionated for the production of PDMP in 2017 (5). Malaysia has been doing contract fractionating with Australia since 1990. Malaysia’s National Blood Centre collected and supplied plasma for the fractionation programme. Factor VIII concentrate, factor IX concentrate, IVIG and albumin were among the fractionated products returned to Malaysia (6).

The administration of IVIG has become an essential therapeutic in clinical medicine for more than 60 years (7). The first immune globulin products made from human plasma were utilised in 1952 by Bruton to treat primary immune deficiency (8). The original use of IVIG was as an antibody replacement treatment. However, numerous other clinical advantages of IVIG therapy, such as immunomodulator, have been observed in many specialities (9). IVIG formulas have improved over time, in addition to production techniques. These products were often freeze-dried in the presence of a stabiliser because IgG solutions at neutral pH are unstable and form precipitates during storage. Given the better stability of IgG solutions at a low pH, current IVIG products are now available as liquids (10).

Immunoglobulin (Ig) demand continues to rise at a regular annual pace of roughly 10%–12% around the world (11). The factors that influence annual consumption growth are complex, including increased use in secondary immunodeficiency diseases, neurological diseases and improved diagnosis for primary immunodeficiencies, especially in developed countries (4).

To date, so far, there is limited data for patients who received IVIG in Malaysia. The absence of a national registry for IVIG usage further compounds this issue. As a result, we lack data on actual IVIG usage among Malaysian patients, making this study necessary.

## Methods

This was a retrospective cross-sectional study involving 482 patients who received IVIG in Hospital Kuala Lumpur, Malaysia, from January 2018 until December 2019. Hospital Kuala Lumpur is one of the biggest government tertiary referral centres in Malaysia and IVIG has been used in Hospital Kuala Lumpur for multi-disciplines. The sample size was calculated using single proportion formula and purposive sampling was performed. The inclusion criteria were all adult and paediatric (age less than 18 years old) patients who received IVIG. Those with incomplete data in requesting forms, or incomplete documentation, were excluded from the study.

Data were extracted from the request form for IVIG, which is recorded in the Pharmacy Department Hospital Kuala Lumpur. All data comprised of patients’ demographics, indications of IVIG and the dosage of IVIG used were analysed. Statistical analysis was performed using IBM SPSS version 27.0 for Windows software (SPSS, Chicago, Illinois, USA). The characteristic of IVIG usage for one-off treatment and regular therapy in adult and paediatric cohorts were analysed and presented descriptively. The categorical data were expressed as frequency (percentage) and numerical data as median (interquartile range [IQR]). The associated factors (age, gender, ethnicity and indications) toward the frequency status (one-off or regular) of IVIG usage were determined using the Pearson’s chi-squared test and independent sample *t*-test. A *P*-value of < 0.05 was considered statistically significant.

## Result

A total of 482 patients in Hospital Kuala Lumpur received IVIG during the study period from 1 January 2018 until 31 December 2019. The median age of patients who received IVIG was 27 years old (IQR 34.9). Among these patients, 243 (50.4%) were female, 228 (47.3%) male and 11 (2.3%) patients gender were undetermined. The highest ethnicity using IVIG were Malay 323 (67.0%) followed by Chinese 82 (17.0%), Indian 51 (10.6%) and others 26 (5.4%). The total amount of IVIG administration was 89.7 kg.

In our study, there were 283 cases of adults and another 199 cases of paediatric. The distribution of indications within each category and the number of patients who received IVIG for each indication are shown in Table 1. The highest indications for IVIG usage among patients were hypogammaglobulinemia and other deficiency states, 127 (26.3%), followed by sepsis, 48 (10%). The list of diseases pooled in other indications included viral myocarditis, pemphigus vulgaris, acute gastroenteritis, neuroblastoma, polymyositis, Creutzfeldt-Jacob disease, Steven Johnson syndrome, neonatal jaundice, Bruton disease, Miller-Fisher syndrome, Canomad syndrome, acute flaccid myelitis, graft heart disease, chronic myeloid leukaemia, ataxia and nephrotic syndrome.

In 263 (54.6%) cases, IVIG was administrated based on the United States Food and Drug Administration (FDA) approval, 217 (45%) were off-labelled and 2 (0.4%) cases were undetermined. The most common indication for FDA approval in both adult and paediatric patients were hypogammaglobulinemia and other deficiency states, 88 (31.1%) and 39 (19.6%). The prescribed leading condition for off-labelled indications was myasthenia gravis (MG), 34 (12%) in adults and sepsis, 38 (19.1%) in paediatric patients. The distribution of indications according to FDA approval is shown in Table 2.

There were 72 (35%) cases of hypogammaglobulinemia and other deficiency states that used IVIG as a one-off treatment in adult patients. At the same time, the highest disease in paediatric was Kawasaki disease (KD), with 28 (20.3%) cases. The highest indication for regular therapy of IVIG among adult patients was chronic inflammatory demyelinating polyneuropathy (CIDP), 18 (23.4%) cases and sepsis, 19 (31.1%) in paediatric patients. The summary of the distribution of indications for one-off treatment and regular therapy is shown in Table 3.

**Figure 1:**
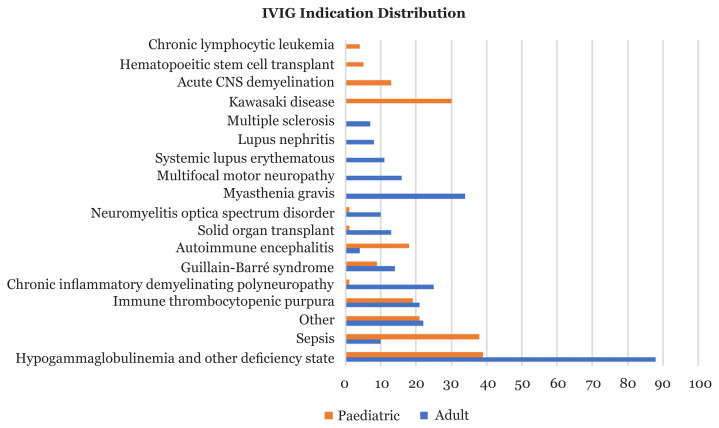
Distribution of IVIG indications between adult and paediatric patients

This study found that the total highest usage of IVIG was hypogammaglobulinemia and other deficiency states in both adult and paediatric patients. However, there were differences in the types of indications between these two groups, with KD and sepsis being the most common in the paediatric cohort in both one-off treatment and regular therapy. In the adult cohort, hypogammaglobulinemia and other deficiency states were most common in one-off treatment, while CIDP was most common in regular therapy. A comparison of IVIG indications between both adult and paediatric patients is shown in Figure 1.

A Chi-squared test was performed to determine factors associated with the frequency status of IVIG usage, including gender, ethnicity and indications. There was no significant association between gender and ethnicity with the frequency status of IVIG usage in both adult and paediatric patients (P = > 0.05). In this study, the clinical category had a significant association for both adult and paediatric patients to the frequency status of IVIG usage with a P-value of P = 0.004 and P = 0.017. A t-test was done to determine the age factor associated with the frequency status of IVIG usage. There was no significant association between age and the frequency status of IVIG usage in adult patients with P = 0.652. However, in paediatric patients, there was a significant association between age and the frequency status of IVIG usage with P = 0.002. The summary of factors associated with the frequency status of IVIG usage in both cohorts is shown in Table 4.

## Discussion

Since 1990, Malaysia has been doing contract fractionation and producing PDMP such as factor VIII concentrate, factor IX concentrate, IVIG and albumin. IVIG is becoming more important as a treatment for a range of medical diseases, not only because of its capacity to combat infection as a replacement therapy but also because of its anti-inflammatory and immunomodulating effects. In this study, we looked at how IVIG was used in one of Malaysia’s largest tertiary institutions, as well as the factors that were associated with IVIG usage frequency. The purpose of this research was to determine the characteristics of IVIG usage in this tertiary institution.

The majority of the participants in this study were Malay, which is representative of Malaysia’s general population distribution. Our finding was similar to a study done in Hospital Universiti Kebangsaan Malaysia (HUKM) where the highest ethnicity was Malay, 65.2% (12). In this study, the percentage of males and females who utilised IVIG was nearly equal. This finding was similar to a study conducted in Israel and Iran, which revealed that the use of IVIG was nearly equal between males and females (8, 13).

The most common reason for IVIG use varies across studies. We divided this study into adult and paediatric categories to look for indications of IVIG usage. Nearly half of the IVIG treatments given through the years 2018–2019 were for off-labelled indications, while in a similar study in a teaching hospital in Tehran with 119 patients, the rate of FDA-approved indications was more than ours (81.5%) (14). Our findings were nearly similar to a few studies that were done in HUKM, Hospital Taiping and the United Arab Emirates (15–17). However, this value is much higher than those reported in the other studies. A study conducted in Singapore over a 10-year period in two public paediatric hospitals reported that IVIG was used for off-labelled indications in only less than 25.0% of cases, while in Qatar, only 22.7% of children received IVIG for off-labelled indications (18, 19). These variations can result from a variety of causes, such as the incidence of disease in different countries, varying patient conditions, and so forth.

Our study revealed that hypogammaglobulinemia and other deficiency states, as well as sepsis, were the most common indications of IVIG usage. This finding differs from those of previous research conducted at other institutions. In two multi-centre trials conducted in Spain and Canada, the most common indication for IVIG treatment was primary immunodeficiency (30.5% and 19.1%, respectively) (20). Other studies in the United States, Canada and Israel found that bone marrow transplantation procedures (50.7%, 15.1% and 24.0%, respectively) and immune thrombocytopenic purpura (ITP) (15.1%, 13.9% and 20.0%, respectively) were the most common indications (20).

Policies have been created in many institutions to monitor and control the IVIG dispensing process. The majority of IVIG was prescribed by the managing doctor at the health facility, but there were no authorised policies governing its use. This helps to explain why numerous illnesses received unnecessary IVIG prescriptions. In comparison with other studies, our finding for the most common clinical condition for off-labelled IVIG usage among adult patients was MG, while in paediatric patients, it was sepsis. These findings were different from a study on 123 paediatric patients in Qatar, where they found the highest usage for off-labelled IVIG usage was in neurological cases (35.4%) (19). A study in the United Arab Emirates found that the highest indication for non-FDA-approved usage in paediatrics was neonatal sepsis (20.0%) and thrombocytopenia (16.0%) in adult patients (17).

Hypogammaglobulinemia and other deficiency states, with 72 (35.0%) cases and MG, with 27 (13.1%) cases, were the most common reasons for one-off treatment in adult patients. CIDP, on the other hand, contributed the most to IVIG regular therapy. The FDA had approved IVIG treatment for hypogammaglobulinemia and CIDP. IVIG is widely used as first-line therapy for inflammatory neurological illnesses such as CIDP and multifocal motor neuropathy (MMN) (14). In addition, IVIG therapy for Guillain-Barré syndrome (GBS) is an off-label indication (14). In terms of therapy preferences, 44% of community neurologists in the United States claimed IVIG was their first choice of treatment for the treatment of CIDP (21). According to the European Academy of Neurology/Peripheral Nerve Society (EFNS), a total IVIG dose of 2 g/kg is routinely administered over 2 to 5 days (22). As not all patients respond to the first course of IVIG, two to five further doses of 1 g/kg IVIg every 3 weeks may be necessary before the patient recovers or IVIg is declared ineffective (22).

Symptomatic medicine, immunomodulators and immunosuppressive agents are used to treat MG (23–25). IVIG was utilised for exacerbation or myasthenic crisis in 24 (70.6%) patients prior to chronic Ig therapy, according to Toronto research (23). However, the role of IVIG in chronic MG therapy has yet to be established. IVIG therapy may be used instead of plasmapheresis for acute MG exacerbations, but it is not suggested for chronic disease management (14).

The most common indications of IVIG usage among paediatric patients in our study were hypogammaglobulinemia and other deficiency states, as well as sepsis. However, this result contrasted with another study. Kawasaki disease (KD) and neonatal sepsis were the most common indications for IVIG, according to a study conducted at HUKM (15). Another study in Singapore found that KD was associated with high IVIG utilisation over a 10-year period (18).

In this study, we discovered that the use of IVIG in KD contributed to the high use of one-off treatment IVIG among paediatric patients. This result was supported by a previous study, which stated that as soon as the diagnosis is confirmed, all patients should receive a single dose of IVIG (2 g/kg) (10). Neutrophil counts, fever and acute-phase reactants are usually reduced within 24 h after treatment (10). A meta-analysis of data from randomised controlled trials of IVIG in KD indicated that a single 2-g/kg dosage of IVIG was linked with a substantial reduction in new coronary artery abnormalities 30 days after diagnosis (26). Patients who got the single infusion performed better, with lower fever, shorter fever duration and a decreased frequency of coronary artery abnormalities, especially in the first 2-weeks (26).

Our research discovered that sepsis was the most common reason for IVIG use as regular IVIG therapy in the paediatric cohort, with the other half of patients receiving IVIG as a one-off treatment. According to a study conducted in Hospital Kuala Lumpur, *Staphylococcus aureus* (17.0%), *Klebsiella pneumoniae* (15.0%), *Acinetobacter baumanii* (10.0%), *Pseudomonas aeruginosa* (10.0%) and *Escherichia coli* (6.0%) were the most common causes of bloodstream infection in paediatric patients. Hospital-acquired infections accounted for the majority of infections (72.0%), followed by healthcare-associated infections (20.0%) and community-acquired infections (8.0%). Methicillin-resistant *Staphylococcus aureus* (MRSA) was found in 33.0% of patients, extended-spectrum beta-lactamase (ESBL) *Klebsiella pneumoniae* was found in 53.0% of cases and ESBL *Escherichia coli* was found in 33% of cases. The overall case fatality rate was 27.0% (27). A meta-analysis of data from eight trials including 492 patients found that adjuvant therapy of bacterial sepsis or septic shock with polyclonal IVIG was linked with considerably lower mortality (10). IVIG, as a supportive therapy in sepsis, has been controversial and not without risk. Serious adverse reactions include the development of a hyper-viscosity syndrome with thromboembolic events in certain patients. Acute renal failure has also been reported, which is thought to be associated with the stabilisers in the IVIG formulations (28). Improvement in serum bactericidal activity by neutralisation and opsonisation of IgG and IgM antibodies, as well as activation of phagocytosis and neutralisation of bacterial toxins, are all likely beneficial mechanisms of IVIG (10). IVIG may also stop endotoxin or superantigen-activated blood cells from releasing proinflammatory cytokines (10). A meta-analysis of 19 studies involving over 1,500 patients found that employing immunoglobulin M (IgM) and immunoglobulin A (IgA) enriched immunoglobulins reduced mortality significantly when compared to human albumin solution or no special therapy as a control intervention (28). However, the ideal dosage, manner of administration, time to start and target value to be obtained while using IgM and IgA enriched immunoglobulin have yet to be determined (28).

Our study found that there was no significant association between demographic characteristics (age, gender and ethnicity) with the frequency status of IVIG usage among adult patients. In paediatric patients, the results were nearly identical. However, there was a significant association between age and the frequency status of IVIG. The clinical category was related to the frequency of IVIG use in both adult and paediatric patients. The FDA had approved seven indications for IVIG usage. Moreover, there were a lot of protocols and guidelines in other countries on the administration of IVIG in the clinical area. Hence, the association between clinical categories compared to the frequency of IVIG usage could be explained by the clinicians following the available guidelines.

Our research had few limitations. It was a single-centre study. As a result, the findings may not be representative of national practices. IVIG has a significant impact on a variety of medical specialities. Despite the fact that Hospital Kuala Lumpur is Malaysia’s largest tertiary centre in Klang Valley; the majority of adult haematological cases are treated at Hospital Ampang (as the designated hospital for adult haematological cases). This might contribute to inconsistency in capturing the IVIG indications for adult haematological cases which are at risk of immunoparesis (e.g. plasma cell disorder, chronic lymphocytic leukemia and haematopoietic stem cell transplantation). The length of hospitalisation and impact on IVIG use were not examined in our study. When standard therapies have failed, become intolerable or are contraindicated, IVIG can be considered only as a second-line therapy. Therefore, future clinical trials must improve, in order to determine the true efficacy of IVIG treatment in certain conditions.

## Conclusion

Given the polarity of immunoglobulin replacement therapy, a national guideline on the prescription of IVIG for patients is urgently needed to assist clinicians in appropriately prescribing IVIG.

## Figures and Tables

**Table 1:** Indications within each category and descriptive analysis of patients who received IVIG

Indication of IVIG usage	*N* = 482 *n* (%)
Immunology	
Hypogammaglobulinemia and other deficiency states	127 (26.3)
Immune thrombocytopenic purpura	40 (8.3)
Autoimmune encephalitis	22 (4.6)
Systemic lupus erythematous	11 (2.3)
Lupus nephritis	8 (1.7)
Neurology	
Myasthenia gravis	34 (7.1)
Chronic inflammatory demyelinating polyneuropathy	26 (5.4)
Guillain-Barré syndrome	23 (4.8)
Multifocal motor neuropathy	16 (3.3)
Acute CNS demyelination	13 (2.7)
Neuromyelitis optica spectrum disorder	11 (2.3)
Multiple sclerosis	7 (1.5)
Medical	
Sepsis	48 (10)
Kawasaki disease	30 (6.2)
Chronic lymphocytic leukaemia	4 (0.8)
Transplant	
Solid organ transplant	14 (2.9)
Hematopoietic stem cell transplant	5 (1.0)
Other	43 (8.9)

Notes: CNS = central nervous system; IVIG = intravenous immunoglobulin

**Table 2:** Descriptive analysis of IVIG indications following FDA approval (labelled and off labelled) in adult and paediatric patients

FDA indication	Labelled		Off labelled	
Adult	***n*** ** = 150**	***n*** ** (%)**	***n*** ** = 133**	***n*** ** (%)**

	Hypogammaglobulinemia and other deficiency states	88 (31.1)	Myasthenia gravis	34 (12.0)
	Chronic inflammatory demyelinating polyneuropathy	25 (8.8)	Other	22 (7.8)
	Immune thrombocytopenic purpura	21 (7.4)	Guillain-Barré syndrome	14 (4.9)
	Multifocal motor neuropathy	16 (5.7)	Solid organ transplant	13 (4.6)
			Systemic lupus erythematous	11 (3.9)
			Sepsis	10 (3.5)
			Neuromyelitis optica spectrum disorder	10 (3.5)
			Lupus nephritis	8 (2.8)
			Multiple sclerosis	7 (2.5)
			Autoimmune encephalitis	4 (1.4)

Paediatric	***n*** ** = 98**	***n*** ** (%)**	***n*** ** = 99**	***n*** ** (%)**

	Hypogammaglobulinemia and other deficiency states	39 (19.6)	Sepsis	38 (19.1)
	Kawasaki disease	30 (15.1)	Other	19 (9.5)
	Immune thrombocytopenic purpura	19 (9.5)	Autoimmune encephalitis	18 (9.0)
	Hematopoietic stem cell transplant	5 (2.5)	Acute CNS demyelination	13 (6.5)
	Chronic lymphocytic leukaemia	4 (2.0)	Guillain-Barré syndrome	9 (4.5)
			Solid organ transplant	1 (0.5)

**Table 3:** Descriptive analysis indications IVIG according to one-off treatment versus regular therapy in adult and paediatric patients

	One-off treatment		Regular therapy	
Adult	***n*** ** = 206**	***n*** ** (%)**	***n*** ** = 77**	***n*** ** (%)**

	Hypogammaglobulinemia and other deficiency states	72 (35)	Chronic inflammatory demyelinating polyneuropathy	18 (23.4)
	Myasthenia gravis	27 (13.1)	Hypogammaglobulinemia and other deficiency states	16 (20.8)
	Immune thrombocytopenic purpura	19 (9.2)	Multifocal motor neuropathy	10 (13.0)
	Other	16 (7.8)	Myasthenia gravis	7 (9.1)
	Guillain-Barré syndrome	13 (6.3)	Solid organ transplant	6 (7.8)
	Systemic lupus erythematous	9 (4.4)	Other	6 (7.8)
	Neuromyelitis optica spectrum disorder	9 (4.4)	Autoimmune encephalitis	3 (3.9)
	Sepsis	9 (4.4)	Multiple sclerosis	3 (3.9)
	Lupus nephritis	7 (3.4)	Immune thrombocytopenic purpura	2 (2.6)
	Chronic inflammatory demyelinating polyneuropathy	7 (3.4)	Systemic lupus erythematous	2 (2.6)
	Solid organ transplant	7 (3.4)	Lupus nephritis	1 (1.3)
	Multifocal motor neuropathy	6 (2.9)	Guillain-Barré syndrome	1 (1.3)
	Multiple sclerosis	4 (1.9)	Sepsis	1 (1.3)
	Autoimmune encephalitis	1 (0.5)	Neuromyelitis optica spectrum disorder	1 (1.3)

Paediatric	***n*** ** = 138**	***n*** ** (%)**	***n*** ** = 61**	***n*** ** (%)**

	Kawasaki disease	28 (20.3)	Sepsis	19 (31.1)
	Hypogammaglobulinemia and other deficiency states	22 (15.9)	Hypogammaglobulinemia and other deficiency states	17 (27.9)
	Sepsis	19 (13.8)	Acute CNS demyelination	8 (13.1)
	Immune thrombocytopenic purpura	18 (13.0)	Hematopoietic stem cell transplant	4 (6.6)
	Autoimmune encepalitis	17(12.3)	Other	4 (6.6)
	Other	17 (12.3)	Kawasaki disease	1 (1.6)
	Guillain-Barré syndrome	8 (5.8)	Immune thrombocytopenic purpura	1 (1.6)
	Acute CNS demyelination	5 (3.6)	Chronic inflammatory demyelinating polyneuropathy	1 (1.6)
	Chronic lymphocytic leukaemia	3 (2.2)	Guillain-Barré syndrome	1 (1.6)
	Hematopoietic stem cell transplant	1 (0.7)	Neuromyelitis optica spectrum disorder	1 (1.6)
			Chronic lymphocytic leukaemia	1 (1.6)
			Solid organ transplant	1 (1.6)
			Autoimmune encephalitis	1 (1.6)

Notes: CNS = central nervous system; IVIG = intravenous immunoglobulin
